# Semisupervised Learning with Report-guided Pseudo Labels for Deep Learning–based Prostate Cancer Detection Using Biparametric MRI

**DOI:** 10.1148/ryai.230031

**Published:** 2023-07-26

**Authors:** Joeran S. Bosma, Anindo Saha, Matin Hosseinzadeh, Ivan Slootweg, Maarten de Rooij, Henkjan Huisman

**Affiliations:** From the Diagnostic Image Analysis Group, Department of Medical Imaging, Radboud University Medical Center, Geert Grooteplein Zuid 10, 6525 GA Nijmegen, the Netherlands.

## Abstract

**Purpose:**

To evaluate a novel method of semisupervised learning (SSL) guided by automated sparse information from diagnostic reports to leverage additional data for deep learning–based malignancy detection in patients with clinically significant prostate cancer.

**Materials and Methods:**

This retrospective study included 7756 prostate MRI examinations (6380 patients) performed between January 2014 and December 2020 for model development. An SSL method, report-guided SSL (RG-SSL), was developed for detection of clinically significant prostate cancer using biparametric MRI. RG-SSL, supervised learning (SL), and state-of-the-art SSL methods were trained using 100, 300, 1000, or 3050 manually annotated examinations. Performance on detection of clinically significant prostate cancer by RG-SSL, SL, and SSL was compared on 300 unseen examinations from an external center with a histopathologically confirmed reference standard. Performance was evaluated using receiver operating characteristic (ROC) and free-response ROC analysis. *P* values for performance differences were generated with a permutation test.

**Results:**

At 100 manually annotated examinations, mean examination-based diagnostic area under the ROC curve (AUC) values for RG-SSL, SL, and the best SSL were 0.86 ± 0.01 (SD), 0.78 ± 0.03, and 0.81 ± 0.02, respectively. Lesion-based detection partial AUCs were 0.62 ± 0.02, 0.44 ± 0.04, and 0.48 ± 0.09, respectively. Examination-based performance of SL with 3050 examinations was matched by RG-SSL with 169 manually annotated examinations, thus requiring 14 times fewer annotations. Lesion-based performance was matched with 431 manually annotated examinations, requiring six times fewer annotations.

**Conclusion:**

RG-SSL outperformed SSL in clinically significant prostate cancer detection and achieved performance similar to SL even at very low annotation budgets.

**Keywords:** Annotation Efficiency, Computer-aided Detection and Diagnosis, MRI, Prostate Cancer, Semisupervised Deep Learning

*Supplemental material is available for this article.*

Published under a CC BY 4.0 license.

## Graphical abstract

**Figure:**
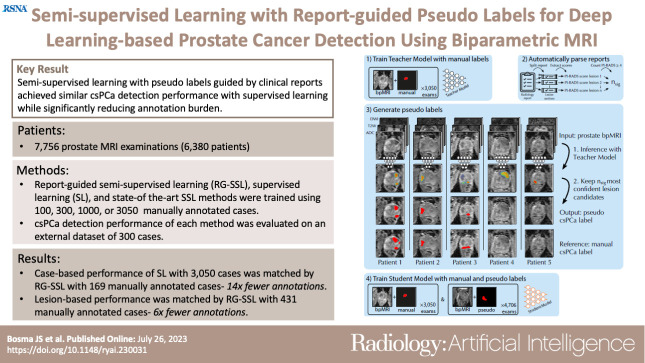

**Summary**

Malignancy detection models trained using semisupervised learning with pseudo labels guided by clinical reports required up to 14 times fewer manual annotations for training and achieved similar performance compared with supervised learning methods.

**Key Points**

■ A novel semisupervised learning (SSL) method, which leverages clinical reports to guide voxel-level pseudo labels, was developed for joint detection and segmentation of malignancy.
■ Report-guided SSL reduced the required number of manual annotations by up to 14 times for detection of clinically significant prostate cancer using biparametric MRI examinations.
■ Report-guided SSL with 100 manually annotated prostate examinations improved area under the receiver operating characteristic curve for risk stratification for clinically significant prostate cancer from 0.78 ± 0.03 to 0.86 ± 0.01 (*P* < .001) and improved lesion-based sensitivity at one false-positive per examination from 48.9% ± 5.0 to 67.1% ± 2.6 (*P* < .001).

## Introduction

Deep learning–based malignancy detection algorithms that perform at the level of clinical experts are typically trained using large, fully annotated datasets (1–3). To allow localization of deep learning malignancy predictions and subsequent interpretability by clinicians, dense voxel-level annotations are required for these datasets. Supervised learning (SL) using large-scale annotation may achieve expert-level performance but is time intensive and costly, especially for dense voxel-level delineations, resulting in substantially smaller labeled training datasets for most malignancy detection use cases. Therefore, it is crucial to reduce annotation burden while achieving optimal performance.

Deep learning with partially missing annotations is effective in the natural image domain, even when manually labeled samples are abundant. On ImageNet, with 1.3 million manually labeled training samples, all 10 leaderboard holders of the past 4 years improved performance by using additional unlabeled data (4–6). In the medical domain, popular techniques to leverage unlabeled data include self-supervised pretraining and semisupervised learning (SSL) with automatically generated pseudo labels or consistency regularization (7,8).

Diagnostic medical reports contain clinical information about the data and are typically available from clinical routine. Use of this clinical information to improve training with unlabeled data is underexplored. Although clinical information from reports typically differs from regular training annotations, it can inform the generation of pseudo labels for SSL. One study (9) generated pixel-level Gleason score annotations in thousands of prostate biopsy specimens by leveraging pathology reports. Bulten et al generated precise cancer masks, to which they assigned the Gleason score extracted from the pathology report. These annotations would have been infeasible to acquire manually. Incorporation of clinical information to guide SSL remains to be investigated for malignancy detection use cases other than biopsy grading.

In general, medical detection tasks in which the structures of interest can be counted might leverage unlabeled examinations by SSL with report-guided pseudo labels. Herein, we focus on lesion detection, wherein each patient can have any number of lesions. To demonstrate the feasibility of our novel method, we developed an SSL method for clinically significant prostate cancer detection using MRI.

Noninvasive diagnosis of clinically significant prostate cancer is crucial to reduce both overtreatment and unnecessary (confirmatory) biopsies (10). Multiparametric MRI scans interpreted by expert prostate radiologists provide the best noninvasive diagnosis (11) but cannot be leveraged freely. Computer-aided diagnosis can help radiologists to diagnose clinically significant prostate cancer, but present-day solutions lack stand-alone performance similar to that of expert radiologists (12–16).

Datasets used for detection and diagnosis of prostate cancer have significantly fewer training samples than datasets used to train top-performing deep learning systems in other medical applications (1–3). For example, Ardila et al (1) used 29 541 training examinations (10 306 patients) for the detection of lung cancer, whereas studies investigating detection of clinically significant prostate cancer with MRI using histopathologically confirmed annotations used deep learning systems trained on 66–806 examinations (median, 146 examinations) (15–22). Approaches using radiologically estimated annotations (reported using the Prostate Imaging Reporting and Data System [PI-RADS] version 2 or 2.1) used 687–1736 training examinations (median, 1584 examinations) (13,14,23–25).

A previous study investigated the effect of training set size on performance of clinically significant prostate cancer detection (14). The authors found that the patient-based area under the receiver operating characteristic curve (AUC) for their internal test set increased logarithmically between 50 and 1586 training examinations from 0.80 to 0.88. If this trend continues, tens of thousands of annotated examinations would be required to reach expert-level performance—in concordance with similar applications in medical imaging (1–3).

According to our principal annotator, about 4 minutes are needed to annotate one prostate cancer lesion on three-dimensional images. Difficult examinations are discussed with radiologists, further increasing the overall duration. Annotating tens of thousands of examinations would therefore incur huge costs and a large time investment, motivating efforts to reduce this annotation burden.

We show that SSL with report-guided pseudo labels can leverage unlabeled examinations to significantly improve detection performance, without additional manual effort. In addition, we show that our method allows development of detection artificial intelligence with similar performance compared with supervised training, while requiring substantially fewer manual annotations. To demonstrate efficacy, we trained semisupervised models for detection of clinically significant prostate cancer with several manual annotation budgets and compared them against supervised training and state-of-the-art semisupervised training methods. Pseudo labels were generated offline, allowing easy integration into any existing training framework.

## Materials and Methods

### Report-guided SSL

**Figure 1:**
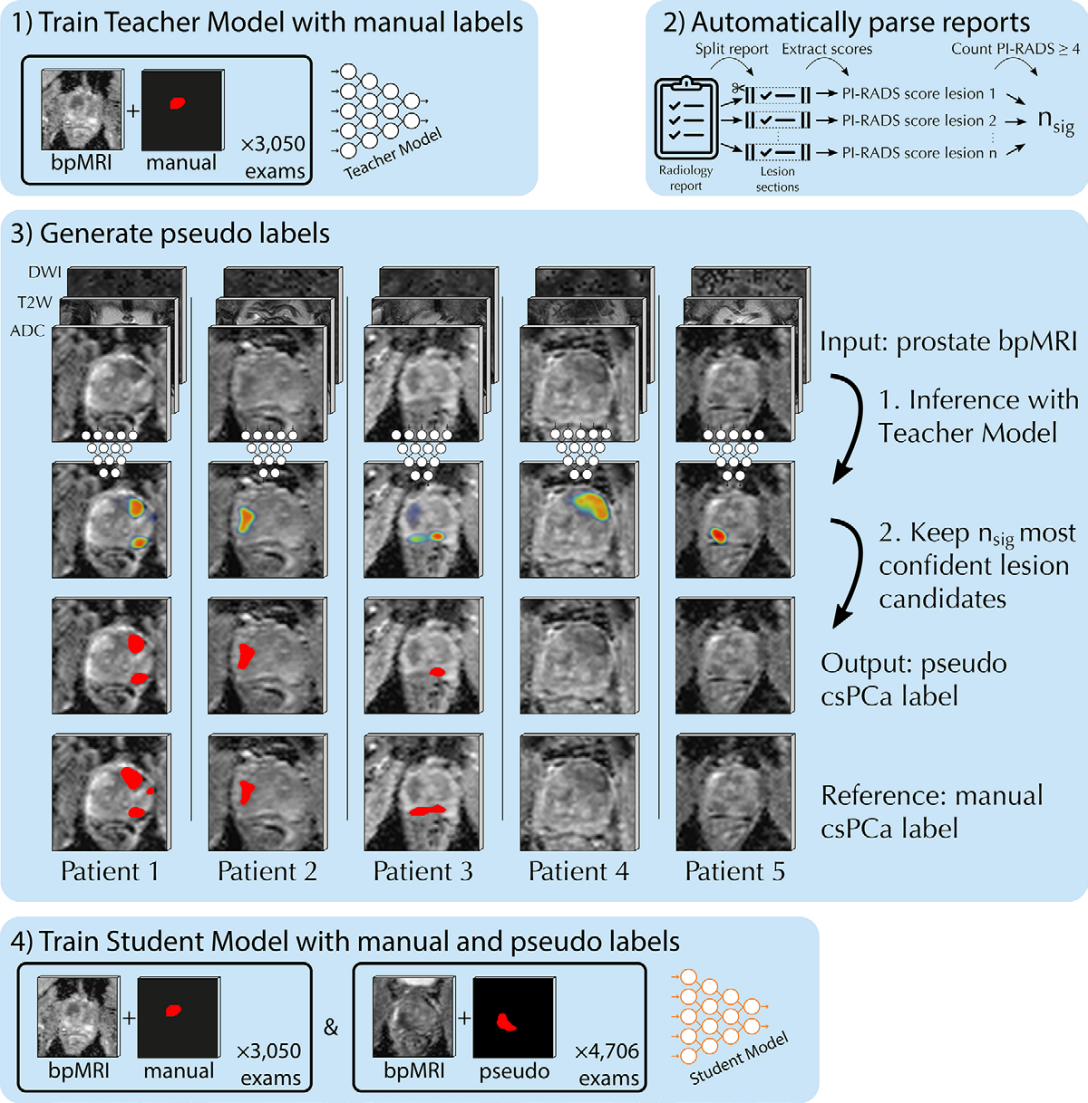
Overview of the semisupervised learning method for malignancy detection: (*1*) train the teacher model with manual labels; (*2*) count the number of clinically significant lesions described in the report, *n*_sig_; (*3*) localize and segment the lesions, by keeping the *n*_sig_ most confident lesion candidates of the teacher model; (*4*) train the student model with manual and pseudo labels. ADC = apparent diffusion coefficient, bpMRI = biparametric MRI, csPCA = clinically significant prostate cancer, DWI = diffusion-weighted imaging, PI-RADS = Prostate Imaging and Reporting Data System, T2W = T2-weighted.

Our novel SSL method leverages diagnostic reports to guide the generation of pseudo labels for semisupervised malignancy detection. At a high level, our report-guided SSL method consists of the following four steps: The first step is to train a supervised model with manually labeled examinations, commonly referred to as the teacher model. The second is to automatically parse the diagnostic reports to assess the number of clinically significant findings in unlabeled examinations, *n*_sig_. The third is to predict the cancer likelihood heatmap for unlabeled examinations with the teacher model and generate pseudo labels by iteratively extracting the *n*_sig_ most likely lesion candidate from the heatmap. The fourth and final step is to train a semisupervised model on the full dataset with manually and automatically labeled examinations, commonly referred to as the student model. Optionally, the student model can be used as the teacher model in a second iteration. The pipeline is depicted in Figure 1 and described in more detail below. The code is publicly available (*https://fastmri.eu/research/bosma22a*).

***Count clinically significant findings in diagnostic reports.—*** Our report-guided pseudo labels leverage the number of clinically significant findings described in the diagnostic report (*n*_sig_). For detection of clinically significant prostate cancer using MRI, we defined *n*_sig_ as the number of lesions deemed (very) likely to harbor clinically significant prostate cancer by the radiologist (PI-RADS ≥ 4). See Appendix S1 for details on the automatic extraction of *n*_sig_ from the radiology reports from clinical routine.

***Generate report-guided pseudo labels.—*** Report-guided pseudo labels were generated in an offline fashion. First, a teacher model was trained on the manually labeled examinations. Then, we performed inference with the teacher model, which comprises an ensemble of clinically significant prostate cancer segmentation models. (Multiple models were ensembled by averaging the softmax confidence maps, which resulted in more consistent segmentation masks compared with a single model. The ensemble also improved localization of report findings in difficult examinations, where a single model was more likely to miss the lesion.) From the resulting voxel-level confidence maps, we created detection maps with distinct lesion candidates, as described in detail in Appendix S1.

Report-guided pseudo labels were then generated by keeping the *n*_sig_ candidates with highest confidence for a lesion. Examinations with fewer lesion candidates than clinically significant report findings were excluded.

### Datasets

Two retrospective datasets with biparametric MRI scans (axial T2-weighted, calculated high-*b*-value [≥1400 sec/mm^2^] diffusion-weighted imaging, and apparent diffusion coefficient maps) for prostate cancer detection were used.

The development dataset (D_dev_) was used to train and tune our models and included 7756 examinations (6380 patients) from 9275 consecutive examinations (7430 patients) performed between January 2014 and December 2020 at Radboud University Medical Center. A total of 1519 examinations were excluded because of incomplete examinations, previous treatment, severe misalignment between sequences, severe artifacts, a previous positive biopsy finding (Gleason grade group [GGG] ≥ 2) (26), or preprocessing errors. See Figure S3 for details. All scans were obtained during clinical routine and were evaluated by at least one of six experienced radiologists (4–25 years of experience with prostate MRI).

The manually labeled development dataset (D_dev,labeled_) comprised the 3050 examinations from D_dev_ performed between January 2016 and August 2018. All 1315 lesions graded as PI-RADS 4 or greater were manually delineated by trained investigators (I.S. and M.H., at least 1 year of experience), who in turn were supervised by an experienced radiologist (M.d.R., 7 years of experience with prostate MRI).

To test our models, an external dataset (test dataset [D_test_]) of 300 examinations (300 patients) performed between March 2015 and January 2017 from Ziekenhuisgroep Twente was used. All patients in the test set underwent transrectal US-guided biopsy, and patients with suspicious findings at MRI (PI-RADS ≥3) also underwent MRI-guided biopsy. For 61 patients (20.3%), radical prostatectomy was performed (see Fig S4 for details). The presence of clinically significant prostate cancer (GGG ≥ 2) was derived from radical prostatectomy (if available) or MRI-guided biopsy. Systematic biopsies were used to upgrade MRI-guided biopsy findings but were not used to downgrade findings. All examinations in the test set had histopathologically confirmed ground truth while retaining the patient cohort observed in clinical practice.

Further details on patient demographic characteristics, examination inclusion or exclusion criteria, and acquisition parameters can be found in Table 1 and Appendix S1. We previously reported on 2436 examinations from D_dev_ and 296 examinations from D_test_ (13) and 2372 examinations from D_dev_ and 293 examinations from D_test_ (14). The current study refined the exclusion criteria and clinical annotations and expanded the datasets with newly curated examinations. Written informed consent was waived by the institutional review board.

**Table 1:**
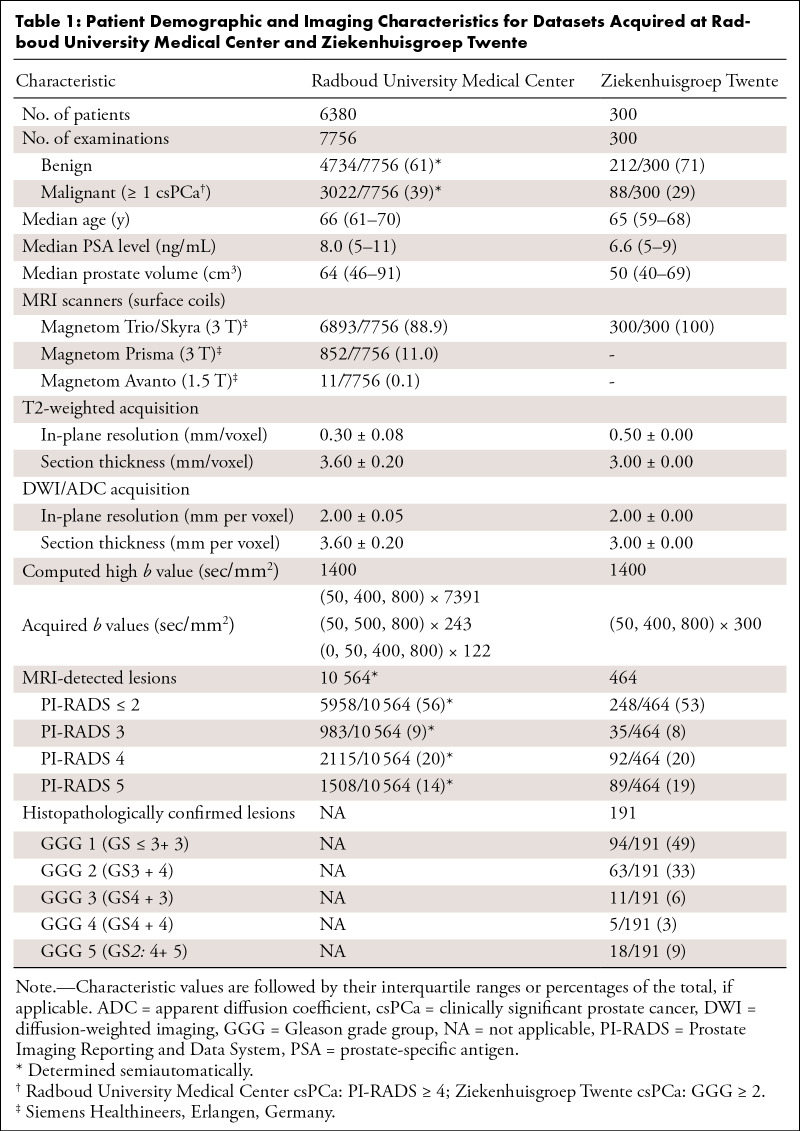
Patient Demographic and Imaging Characteristics for Datasets Acquired at Radboud University Medical Center and Ziekenhuisgroep Twente

### Models, Preprocessing, and Data Augmentation

We posed the prostate cancer detection task as a voxel-level segmentation task and used the nnU-Net framework. The nnU-Net is a self-configuring framework that follows a set of rules to select the appropriate architecture, data augmentations, preprocessing method, and more (Appendix S1) (27).

### SSL Approaches

For the SSL setting, we investigated budgets of 100, 300, 1000, and 3050 manually labeled examinations, paired with the remaining 7656, 7456, 6756, and 4706 unlabeled examinations, respectively. Models were trained with fivefold cross-validation, with randomly generated cross-validation splits at the patient level.

For report-guided SSL, the teacher model was used to generate pseudo labels for the unlabeled portion of the training data by ensembling the predictions of the 15 models (three restarts, fivefold cross-validation). The pseudo-labeled data were combined with the manually labeled data to train a student model. To investigate convergence of our SSL method, a second iteration was performed. The student model from the first iteration then became the teacher model in the second iteration.

Our report-guided SSL was compared with two state-of-the-art SSL methods for medical image segmentation without report guidance: uncertainty-aware mean teacher (28) and cross pseudo supervision (Appendix S1) (29).

### Experimental Analysis

First, we evaluated the quality of our report-guided pseudo labels by comparing them with the manual labels. Then, we trained semisupervised student models with several manual annotation budgets. Finally, we calculated the annotation burden reduction.

***Extraction of report findings.—*** The accuracy of natural language processing for automatically counting the number of PI-RADS of 4 or greater lesions in a report (*n*_sig_) was determined by comparing against the number of PI-RADS 4 or greater lesions in D_dev,labeled_. To account for multifocal lesions (which can be annotated as two distinct regions or a single larger one) and human error in the ground truth annotations, we manually checked the radiology report and verified the number of lesions when there was a mismatch between the manual label and automatic estimation.

***Localization of report findings.—*** Localization performance of the artificial intelligence methods was compared using free-response receiver operating characteristic (FROC) analysis. Analysis was performed with fivefold cross-validation on D_dev,labeled_.

Pseudo labels from uncertainty-aware mean teacher and cross pseudo supervision were generated at each training site. For fair comparison, we evaluated the pseudo labels from the best checkpoint (see Appendix S1 for the model selection method).

***Segmentation of report findings.—*** Quality of the correctly localized report findings was evaluated with the Dice similarity coefficient (DSC). This evaluation was performed with fivefold cross-validation on D_dev,labeled_.

To enable spatial similarity evaluation of the soft pseudo labels from uncertainty-aware mean teacher, we binarized the labels with a threshold of 0.5, following the strategy used by cross pseudo supervision.

***Prostate cancer detection.—*** Prostate cancer detection models were evaluated on 300 external examinations with histopathologically confirmed ground truth (D_test_). Examinations with at least one clinically significant prostate cancer (GGG ≥ 2) lesion were considered positive.

Patient-based diagnostic performance was analyzed using receiver operating characteristic (ROC) analysis and was summarized using the AUC. Lesion-based detection performance was analyzed using FROC analysis and was summarized using the partial AUC between 0 and 1 false-positive findings per examination.

***Annotation burden reduction.—*** SSL can leverage unlabeled examinations for training, potentially reducing the number of manually labeled examinations required to reach expert-level diagnostic performance. To investigate the extent to which SSL reduces the annotation burden, we assessed how many manually labeled examinations are required in the semisupervised setting to match the performance achieved in the fully supervised setting with 3050 manually labeled examinations. The annotation burden reduction factor is then defined as follows: 
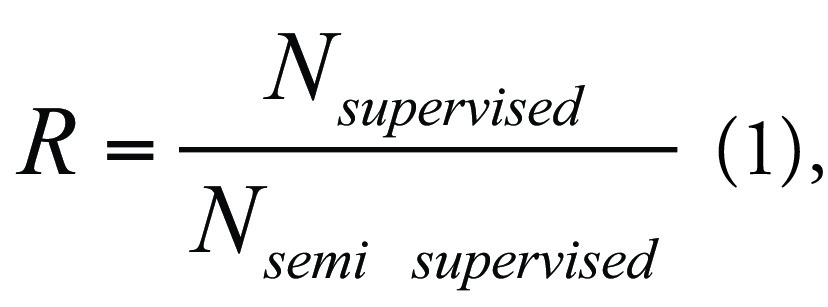
 with *N_supervised_* representing the number of manually labeled examinations used for supervised training and *N_semi supervised_* representing the number of manually labeled examinations used for semisupervised training.

We used piecewise logarithmic interpolation to obtain a continuous performance curve as a function of the number of manually labeled examinations.

### Statistical Analysis

We trained models with fivefold cross-validation and three restarts for our report-guided SSL method, two restarts for uncertainty-aware mean teacher, and one restart for cross pseudo supervision, resulting in 15 or 10 AUCs and partial AUCs on the test set for each model configuration. Comparison of performance between groups of independent models allows for investigation of the difference in performance due to training configuration rather than variation in performance inherent to the stochastic nature of deep learning. (Sources of variation include the model’s random initialization, order of training batches, and data augmentations, resulting in differences in model performance between training runs.) To determine the probability of one configuration outperforming another, we performed a permutation test of the performance metrics with 1 000 000 iterations. We used a statistical significance threshold of .05.

We estimated 95% CIs for the performance of radiologists by bootstrapping 1 000 000 iterations, with each iteration selecting *n*-of-*n* patients with replacement and calculating the target metric. Iterations that sampled only one class were rejected. Statistical analyses were implemented in Python 3.8.

## Results

### Extraction of Report Findings

Our natural language processing score extraction algorithm identified the correct number of PI-RADS 4 or greater lesions for 3024 of the 3044 (99.3%) radiology reports in D_dev,labeled_. Examinations with negative results (PI-RADS ≤3) were identified with 99.7% accuracy.

**Figure 2:**
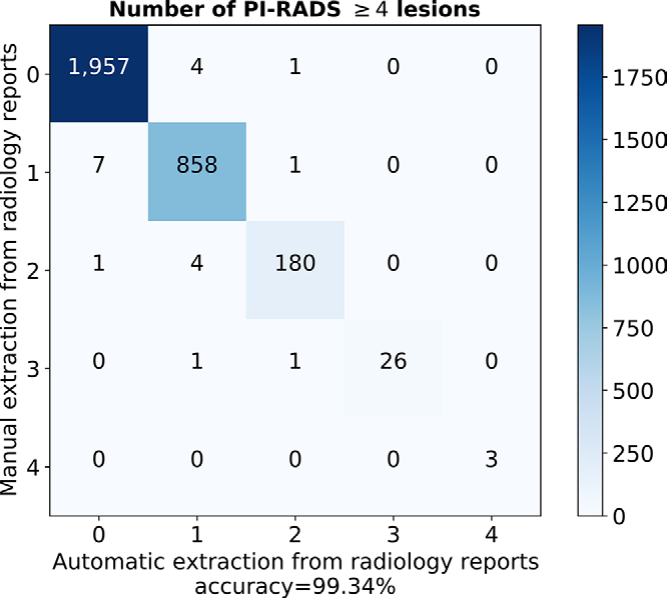
Accuracy of natural language processing score extraction algorithm, as depicted by the confusion matrix for number of clinically significant findings in a radiology report. Evaluated on the manually labeled development dataset. PI-RADS = Prostate Imaging and Reporting Data System.

We excluded reports and their examinations when no PI-RADS scores could be extracted from the report: six of 3050 examinations (0.2%) from D_dev,labeled_ and 121 of 4706 examinations (2.6%) from the remaining examinations of D_dev_ (ie, unlabeled examinations). Manual inspection revealed that 34 of these 121 unlabeled examinations (28%) contained PI-RADS 4 or greater classifications (with nonstandard reporting) or were not a prostate cancer detection examination. Exclusion of these examinations improved the quality of the pseudo-labeled examinations because they would have been included as negative otherwise. Figure 2 shows the full breakdown of automatically extracted versus manually determined number of significant lesions. Typing mistakes and score updates in the addendum were the main source of the 20 (0.7%) incorrect extractions, which is an error rate similar to that observed for our annotators.

### Localization of Report Findings

**Figure 3:**
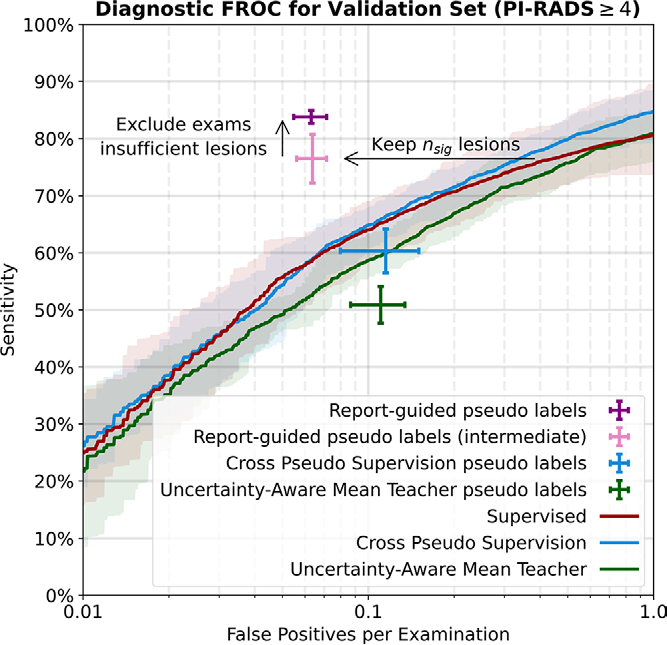
Quality of the pseudo labels, as evaluated by free-response receiver operating characteristic (FROC) analysis for matching manually annotated Prostate Imaging and Reporting Data System (PI-RADS) 4 or greater lesions in the manually labeled development dataset. Supervised models used to generate report-guided pseudo labels were trained with fivefold cross-validation on the manually labeled development dataset. Uncertainty-aware mean teacher and cross pseudo supervision models were trained with fivefold cross-validation on the development dataset. Filtering pseudo labels using the number of clinically significant findings described in the diagnostic report (*n*_sig_) greatly reduced the number of false-positive lesions per examination (report-guided pseudo labels [intermediate]). Excluding examinations with fewer than *n*_sig_ lesion candidates improved sensitivity (report-guided pseudo labels). Shaded areas indicate 95% CIs. Error bars indicate SDs.

The clinically significant prostate cancer detection models achieved high sensitivity. At this high sensitivity, the models also propose many false-positive lesion candidates (Fig 3). Masking the lesion candidates from the teacher model with the number of clinically significant report findings, *n*_sig_, greatly reduced the mean number of false-positive lesions per examination from 0.39 ± 0.14 (SD) to 0.064 ± 0.008 for our report-guided pseudo labels (with fivefold cross-validation on *D*_dev,labeled_). This sixfold reduction in false-positive lesions greatly increased the quality of the pseudo labels.

Examinations for which we could extract fewer than *n*_sig_ lesion candidates were excluded. Among the excluded examinations were those where we are certain to have missed lesions, thus increasing sensitivity. From the first iteration of report-guided pseudo labels, we excluded 119 examinations, resulting in a sensitivity of 83.8% ± 1.1 (192 ± 12 of 229 ± 14 lesions) at 0.063 ± 0.008 (36 ± 4 of 578 ± 15) false-positive lesions per examination across fivefold cross-validation.

Binarization of uncertainty-aware mean teacher–generated soft pseudo labels yielded pseudo labels with a sensitivity of 50.1% ± 3.5 (132 ± 17 of 263 ± 16) at 0.114 ± 0.031 (70 ± 19 of 610 ± 21) false-positive lesions per examination.

Use of cross pseudo supervision generated binarized pseudo labels with a sensitivity of 60.3% ± 3.8 (157 ± 6 of 263 ± 16) at 0.115 ± 0.035 (62 ± 20 of 610 ± 21) false-positive lesions per examination. Binarization of the softmax predictions gave lower detection performance than the FROC curve because our lesion extraction (Appendix S1) performed better than naive binarization. See Figure 3 for an overview of the pseudo label localization quality.

### Segmentation of Report Findings

Spatial similarity between the pseudo and manual labels was good. When trained with fivefold cross-validation on D_dev,labeled_, our report-guided pseudo labels achieved a DSC of 0.67 ± 0.19. When trained semisupervised with fivefold cross-validation on *D*_dev_, pseudo labels from uncertainty-aware mean teacher achieved a DSC of 0.64 ± 0.20, and pseudo labels from cross pseudo supervision achieved a DSC of 0.68 ± 0.19.

Figure 1 shows report-guided pseudo labels, with a DSC of 0.70 (approximate mean) for the upper lesion of patient 1, a DSC of 0.87 (approximate mean + 1 SD) for patient 2, and a DSC of 0.55 (approximate mean − 1 SD) for patient 3.

The full distribution of DSC against lesion volume is given in Appendix S1.

### Detection of Clinically Significant Prostate Cancer

Report-guided SSL significantly increased model performance for all investigated manual annotation budgets compared with SL with the same number of manually labeled examinations (*P* < .001 for each comparison). Iteration 2 generally performed better than iteration 1, although only examination-based performance for manual annotation budgets of 100 or 300 examinations improved significantly (*P* = .004 and *P* = .003, respectively), showing quick convergence. Uncertainty-aware mean teacher and cross pseudo supervision failed to improve model performance compared with SL (all comparisons *P* > .05). The exception was uncertainty-aware mean teacher with 1000 manual labels, in which uncertainty-aware mean teacher outperformed SL at examination-based diagnosis (*P* = .004) and lesion-based detection (*P* < .001).

Report-guided SSL (iteration 2) outperformed uncertainty-aware mean teacher and cross pseudo supervision at examination- and lesion-based performance for all manual annotation budgets (all comparisons *P* < .003), except for examination-based performance of uncertainty-aware mean teacher with 1000 manual labels.

**Figure 4:**
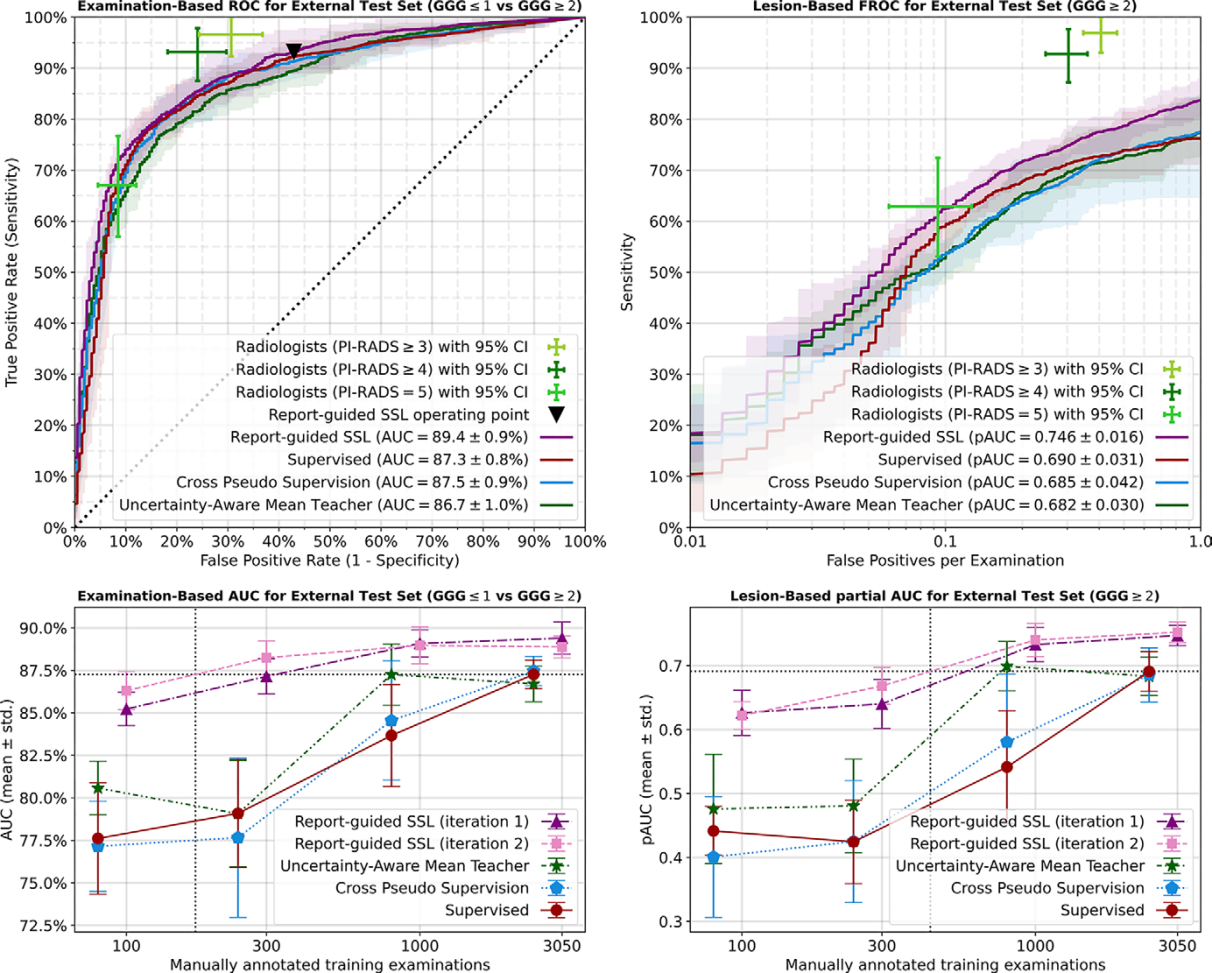
Model performance for semisupervised and supervised learning. Top row: Supervised models were trained with fivefold cross-validation on 3050 manually labeled examinations, and semisupervised learning (SSL) also included 4706 unlabeled examinations. Report-guided SSL significantly outperformed supervised learning as well as the baseline SSL methods. Bottom row: Model performance for 100, 300, 1000, and 3050 manually labeled examinations, combined with 7656, 7456, 6756, and 4706 unlabeled examinations, respectively. Report-guided SSL significantly outperformed the baseline SSL methods and supervised learning at each annotation budget, except for examination-based area under the receiver operating characteristic curve (AUC) of uncertainty-aware mean teacher trained with 1000 labeled examinations. Left: Receiver operating characteristic (ROC) performance for examination-based diagnosis of examinations with at least one lesion with Gleason grade group (GGG) 2 or greater. Right: Free-response ROC (FROC) performance for lesion-based diagnosis of lesions with GGG 2 or greater. All models were trained with radiology-based Prostate Imaging and Reporting Data System 4 or greater labels and evaluated on the external test set with histopathologically confirmed ground truth. Shaded areas indicate the 95% CIs from 15 or five independent training runs. Error bars indicate SDs across 15 or five independent training runs. pAUC = partial area under the receiver operating characteristic curve.

Figure 4 (bottom row) shows a full overview of diagnostic and detection performance for each manual annotation budget. Table 2 shows the AUC values for each configuration. To investigate the dependence of our report-guided SSL method on the nnU-Net training framework, we also investigated the training framework from Saha et al (13). Results for this are shown in Appendix S1.

**Table 2:**
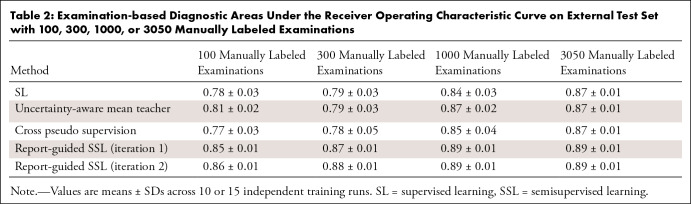
Examination-based Diagnostic Areas Under the Receiver Operating Characteristic Curve on External Test Set with 100, 300, 1000, or 3050 Manually Labeled Examinations

### Annotation Burden Reduction

Report-guided SSL (iteration 2) with 300 manual labels exceeded examination-based AUC performance of SL with 2440 manually labeled examinations. Performance with 100 manual labels came close to SL. Interpolation suggests that supervised performance is matched with 169 manual labels (14 times annotation burden reduction).

Report-guided SSL (iteration 2) with 1000 manually labeled examinations exceeded lesion-based partial AUC performance of SL with 2440 manually labeled examinations. Performance with 300 manual labels came close to that of SL. Interpolation suggests that supervised performance is matched with 431 manual labels (six times annotation burden reduction).

## Discussion

Large-scale SL can reach expert-level diagnostic performance but requires labor-intensive manual annotation, which is expensive and infeasible to obtain for each (cancer) detection use case. Our novel report-guided SSL method significantly improved diagnostic performance at all investigated manual annotation budgets compared with SL and SSL without report guidance except for examination-based performance of uncertainty-aware mean teacher with 1000 manual annotations. The report-guided pseudo labels are of sufficient quality to improve semisupervised malignancy detection, even when only 100 manually labeled examinations are available. This improved performance demonstrates the feasibility of report-guided SSL for malignancy detection.

In this study, the training procedure with report-guided pseudo labels is presented for detection of clinically significant prostate cancer with MRI using radiology reports. However, the underlying method is not limited to clinically significant prostate cancer, MRI, or radiology reports and can be applied universally. Any detection task with countable structures of interest and clinical information reflecting these findings can use our training method to reduce the annotation burden.

Report-guided SSL allowed us to leverage the full dataset of 7756 examinations, which is a substantial increase in the number of training examinations compared with the previous largest dataset for detection of clinically significant prostate cancer using MRI of 1736 examinations. This brings detection of clinically significant prostate cancer much closer to the dataset sizes used to train top-performing deep learning systems, where up to 29 541 training examinations were used.

Negative examinations were identified with 99.7% accuracy, suggesting that negative examinations (approximately 60% of all examinations for detection of clinically significant prostate cancer) can be automatically annotated almost perfectly, speeding up the manual annotation process substantially. Furthermore, the segmentation masks are often of sufficient quality to require only verification of the location, saving ample time for positive examinations as well.

To our knowledge, we are the first to investigate SSL for malignancy detection using three-dimensional images. Our presented method, report-guided SSL, has several limitations that should be considered. Report-guided SSL has the risk of introducing systematic pseudo label errors by reinforcing possibly incorrect model predictions. This could drive the subsequent semisupervised detection model to confidently predict benign abnormalities as being malignant. Thus, careful evaluation of the model before is necessary.

Direct applicability of the rule-based PI-RADS score extraction from radiology reports is limited because it needs to be adapted for reports with different structures or languages. For unstructured reports, a deep learning–based NLP model can be trained on the manually labeled subset to perform the task of counting the number of clinically significant findings.

In addition, PI-RADS 4 or greater lesions reported with PI-RADS version 2 or version 2.1 were used to train the clinically significant prostate cancer detection models. Radiologically estimated lesions contain both false-positive and false-negative lesions. Inclusion of PI-RADS 3 lesions as clinically significant prostate cancer to train the algorithms would decrease the number of false-negative lesions at the cost of introducing many false-positive results. Although these training annotations are not perfect, they have been shown to be suitable for training clinically significant prostate cancer detection models (13,14), as also demonstrated by the evaluation on the test set with histopathologically confirmed lesions. Changes between PI-RADS versions 2 and 2.1 did not affect the classification of PI-RADS 4 and 5 lesions, so this difference in reporting did not affect our training dataset. The exclusion of PI-RADS 3 lesions from the training dataset may cause bias in the algorithms and lead them to suppress these lesions. Subset analysis of patients with PI-RADS 3 lesions should therefore be performed before deployment in clinical practice.

In conclusion, report-guided SSL allows for substantial reduction in annotation burden by leveraging unlabeled examinations paired with diagnostic reports. Our proposed method is widely applicable, paving the way for larger datasets with equal or reduced annotation time.
